# Side-to-side differences in knee laxity and side hop test may predispose an anterior cruciate ligament reinjury in competitive adolescent alpine skiers

**DOI:** 10.3389/fspor.2022.961408

**Published:** 2022-10-14

**Authors:** Maria Westin, Lisbeth I. Mirbach, Marita L. Harringe

**Affiliations:** ^1^Department of Molecular Medicine and Surgery, Stockholm Sports Trauma Research Center, Karolinska Institutet, Stockholm, Sweden; ^2^Aleris Sports Medicine and Ortopedi, Sabbatsbergs Hospital, Stockholm, Sweden; ^3^The Swedish School of Sports and Health Sciences, Stockholm, Sweden

**Keywords:** ACL injury, reinjury, alpine skiing, competition, adolescent, ski high school

## Abstract

An anterior cruciate ligament (ACL) injury is a common, severe injury in alpine skiing, and anterior cruciate ligament reconstruction (ACLR) is frequently performed in competitive alpine skiers younger than 20 years old. To reduce the reinjury rate, both intrinsic and extrinsic risk factors should be examined. The aim of this study was to investigate possible intrinsic risk factors for an ACL reinjury in competitive alpine skiers. A cohort of 384 alpine skiers (191 males/193 females) from the Swedish ski high schools were prospectively followed during their high school years. The students were clinically examined and physically tested prior to each ski season. In addition, the RAND 36-Item health survey 1.0 (SF-36, Copyright 1994 Medical Outcome Trust, distributed by RAND Corporation) and injuries were prospectively registered. Thirty-one of the skiers (five males/26 females) had undergone an ACLR before entering the ski high school. This cohort was analyzed with respect to the occurrence of, and possible risk factors for an ACL reinjury (including ipsilateral and contralateral ACL injuries). Skiers who sustained an ACL reinjury were called the “ACL reinjury group,” and those who did not sustain an ACL reinjury were called the “ACL injury group.” Notably, 12 of the 31 students (39%), ten female and two male skiers, aged 16.5 (SD 0.5) years, sustained an ACL reinjury during the two first years at the ski high school. In addition, 10 of the 12 ACL reinjuries occurred within 10–23 months from the first injury [m 14.8 (SD4.7)] and two ACL reinjuries occurred at 29 and 47 months, respectively, from the first injury. It is noted that eight of the ACL reinjuries were to the ipsilateral knee and four to the contralateral knee. There were no differences between the groups with respect to muscle flexibility in the lower extremity, Beighton score, and one leg hop for distance or square hop test. Side-to-side differences were found with respect to knee joint laxity, >3 mm, measured with KT-1000 arthrometer (*p* = *0.02*), and the side hop test (*p* = *0.04*). RAND 36-Item health survey did not predict an ACL reinjury. In conclusion, a side-to-side difference in the side hop test and knee joint laxity (KT-1000) may predispose an ACL reinjury in competitive adolescent alpine skiers.

## Background

Competitive alpine skiing is an injury-risk sport, and the reports on injury incidence vary depending on how and when the incidence is reported. At the World Cup 2006/2007 and 2007/2008, the injury incidence was 36.7 injuries/100 alpine skiers (1), and at the Olympic games in Sochi, the injury incidence was 20.7 injuries/100 alpine skiers (2). An investigation by Fröhlich et al. (3) studying overuse and traumatic injuries in Swiss elite level skiers over one season showed an incidence of 95.5 injuries/100 alpine skiers. In high school competitive alpine skiers, the incidence of traumatic injuries was reported at 3.11 injuries/100 months attending a ski high school, and it was concluded that half of all the students were at risk for an injury during their 3–4 years of the study period (4). Around one-third of all alpine ski injuries are to the knee and the injuries on average generate more than 28 days of absence from the sport (1, 4). One of the most severe and common injuries to the knee is an anterior cruciate ligament (ACL) injury (5, 6). In a study by Pujol et al. (5) on 379 adult elite French alpine skiers over a period of 25 years, an incidence of 8.5 ACL injuries/100 competitive alpine skiers and season was shown. A total of 157 ACL injuries were registered, and half of the ACL-injured alpine skiers sustained an ACL reinjury to the ipsilateral or the contralateral knee (5). In another study, Stevenson et al. (7) described that 22% of alpine skiers underwent a second ACL reconstruction (ACLR). The general opinion is that an ACLR is necessary to be able to continue with alpine skiing and is frequently performed in young competitive alpine skiers.

To reduce the ACL reinjury rate, both intrinsic and extrinsic risk factors should be examined. Side-to-side differences, decreased core stability, and parents' history of an ACL injury are intrinsic factors mentioned as risk factors for the first ACL injury in alpine skiing (8–10). Risk factors for an ACL reinjury in football seem to be injury events close to previous ACLR (11) or time to return after an ACLR (12) as well as whether it was a contact or non-contact ACL injury the first time. Non-contact injuries and isolated ACL injuries were injury risks for an ACL reinjury in professional footballers (13). Another risk for an ACL reinjury seems to be a young age. Paterno et al. (14) showed that 25% of the athletes returning to pivoting sports, and under the age of 25 years, sustained an ACL reinjury. In a 5-year follow-up from the Swedish National Knee Ligament Register (SNKLR), it was shown that athletes under the age of 20 years at the first ACLR had an almost three-fold risk for a contralateral ACL injury to occur (15). A review on ACL injuries showed that close to one of four athletes (23%) under the age of 25 years, returning to high-level sports with an ACLR, sustained an ACL reinjury (16).

To recover and return to sports after an ACL injury is difficult, and the perfect algorithm is still not known. Biological healing and functional performance are keys to success (17). Another key is mental health and psychological readiness to return to play (18, 19).

To the best of our knowledge, studies on risk factors for sustaining an ACL reinjury in young alpine skiers are still lacking. Therefore, the overall aim of this study was to investigate possible intrinsic risk factors for an ACL reinjury in young competitive alpine skiers during their ski high school years. The second aim was to describe this sub-cohort of students from a health perspective using the RAND 36-Item health survey 1.0 (SF-36, Copyright 1994 Medical Outcome Trust).

## Methods

A cohort of 384 alpine ski students (191 males and 193 females), from ten different Swedish ski high schools, were prospectively followed from September 2006 to May 2009 with respect to performance and injury incidence. A ski student attends high school for 3 or 4 years and is exposed to approximately 200 days of skiing per year (4). The students were included in their first year at the ski high school, and this study was approved by the Swedish Ethical Review Authority, Dnr 2006/833-31/1. Oral and written consent was collected from the ski students.

Of the included 384 ski students, 31 students (five males and 26 females) had undergone an ACLR before entering the ski high school (Table 1). This subgroup of skiers was followed during their studies at the ski high school and in this study occurrence of an ACL reinjury, and possible risk factors for sustaining an ACL reinjury during the ski high school years were analyzed. In this study, the group with skiers sustaining an ACL reinjury is called the “ACL reinjury group” and includes an injury to the ipsilateral or contralateral ACL. The group of skiers that did not sustain an ACL reinjury during their high school years is called the “ACL injury group.”

**Table 1:** Demographic data of the 31 ski high school students who entered the study with a previous anterior cruciate ligament reconstruction (ACLR).

**ACLR at school start**		**Male ski students** ***n =* 5**	**Female ski students** ***n =* 26**
Age	m (SD)	16 (0)	16.3 (0.5)
Height (cm)	m (SD)	175.2 (4.9)	166.3 (5.2)
Weight (kg)	m (SD)	72.4 (9.9)	63.3 (7.1)
Body mass index (BMI)	m (SD)	23.5 (2.6)	22.9 (2.0)
ACL injury left knee	*n*	5	14
ACL injury right knee	*n*	0	11
ACL injury bilateral	*n*	0	1

m, mean value; SD, standard deviation; n, number.

All ski students were clinically examined and physically tested prior to each ski season according to a specific protocol previously described by Westin et al. (8). The protocol included muscle flexibility of hamstrings, rectus femoris, iliopsoas, gastrocnemius, soleus (8), general joint laxity measured with Beighton score (20), knee joint laxity measured with KT-1000 arthrometer (21), and functional performance tests such as the one leg hop for distance, side hop test, and square hop test (22–24). General joint laxity, the Beighton score, was classified as yes (≥ 5 of nine positive tests) and no (≤ 4 positive tests). The skiers also answered the RAND 36-Item health survey 1.0 (25) (SF-36, Copyright 1994 Medical Outcome Trust), and ACL injuries were prospectively registered.

The items in the RAND 36-Item health survey consist of 36 questions regarding physical functioning (10 questions), bodily pain (two questions), role limitations due to physical health problems (four questions), role limitations due to emotional problems (three questions), emotional wellbeing (five questions), social functioning (two questions), energy/fatigue (four questions), general health perceptions (five questions), and at last a single question regarding the perceived change in health since last year. The questions were presented in a mixed order and adapted from a more extensive instrument used in the Medical Outcomes Study (MOS) (26). The scoring system is a two-step process where all the questions in the survey are evaluated on a scale from 0 to 100, and thereafter, the questions that answer each of the eight plus one domain are summarized and averaged. When summarized, 0 represents poor health and 100 represents good health. For more information, visit RAND Corporation (https://www.rand.org/health-care/surveys_tools/mos/36-item-short-form/scoring.html).

### Statistics

Data were presented descriptively with mean values and standard deviation (m(SD)) or with median and interquartile (md(IQ)) or total range (md(range)). Chi-squared test was used analyzing general joint laxity, and the Mann-Whitney *U*-test was used comparing side-to-side differences in the preseason tests between the “ACL reinjury group” and the “ACL injury group” (27). The RAND 36-Item health survey 1.0 (SF-36, Copyright 1994 Medical Outcome Trust) was recorded and analyzed according to recommendations by RAND Corporation, and the eight domains were presented with md (total range) for both groups. The preseason measurements prior to the ACL reinjury season were used for the “ACL reinjury group,” and the preseason measurements from year 2 in the “ACL-injury group” served as control values, though in six cases, the preseason tests from year 1 were used due to missing values in year 2. The *p*-value was set at ≤ 0.05.

## Results

Out of 31, 12 (39%) students, ten female and two male skiers, m 16.5 (SD 0.5) years old, sustained an ACL reinjury. The injuries occurred on average 18.7 months (SD 10.8, range 10–47) from the first ACL injury. Notably, 10 of the 12 ACL reinjuries occurred within 10–23 months from the first injury [m 14.8 (SD 4.7)], and two of the ACL reinjuries occurred 29 and 47 months, respectively, after the first ACL injury. Six ACL reinjuries occurred during the first year at the ski high school (two males/four females) and six ACL reinjuries occurred during the second year (six females). Eight of the ACL reinjuries were to the ipsilateral knee and four reinjuries to the contralateral knee. Ten of the ACL reinjuries were to the left knee and two reinjuries to the right knee. One male skier reinjured his left ACL, and the other male skier injured his right knee, which was the contralateral ACL. Six female skiers reinjured their left ACL, three female skiers injured their contralateral knee of whom two injured their left ACL, and one injured her right ACL (Table 2).

**Table 2:** Alpine ski high school students who sustained an ACL reinjury.

		**Male** ***1st ACL-injury***	**Male** ***2nd*** ***ACL-injury***		**Female** ***1st ACL-injury***	**Female** ***2nd ACL-injury***	
Left knee	n	2	1		7	8	
Right knee	n		1		3	2	
Months between 1st and 2nd injury	m (SD) range			12 (1.4) 11–13			20 (11.4) 10–47

Injured knee at 1st and 2nd injury and the average number of months between the two injuries (*n* = 12). n, number; m, mean value; SD, standard deviation; range (min-max).

There were no differences between the “ACL reinjury group” and the “ACL injury group” with respect to muscle flexibility in hamstrings, rectus femoris, iliopsoas, gastrocnemius, or soleus measured according to the protocol presented by Westin et al. (8), or with respect to general laxity measured with Beighton score, or in the functional performance tests, one leg hops for distance or square hop test. Significant differences were found with respect to knee joint laxity measured with KT-1000 max manual where >3 mm is considered a side-to-side difference and the side-to-side differences in the side hop test. Skiers in the “ACL reinjury group” had a side-to-side difference in knee laxity of md 4 mm (IQ 3–5) compared with skiers in the “ACL injury group” with a side-to-side difference of md 1 mm (IQ 0–3) (*p* = *0.02*). In the side hop test, the side-to-side difference was md three hops (IQ 3–4) in the “ACL reinjury group” compared with md two hops (IQ 1–3) in the “ACL injury group” (*p* = *0.04*). The RAND 36-Item health survey did not predict an ACL reinjury (Table 3).

**Table 3:** Skiers who answered the RAND 36-Item health survey at the start of the preseason tests used in this study.

	**Skiers who answered the health survey** ***n** = **29***	
**RAND 36-Item health survey**	**“ACL reinjury”** ***n = 11***	**“ACL injury”** ***n = 18***
Physical functioning	100 (85–100)	97.5 (77.8–100)
Role limitations due to physical health	100 (25–100)	100 (0–100)
Role limitations due to emotional problems	100 (66.7–100)	100 (66.7–100)
Energy / fatigue	70 (50–100)	75 (40–100)
Emotional wellbeing	80 (64–100)	84 (52–100)
Social functioning	100 (75–100)	100 (37.5–100)
Pain	77.5 (47.5–100)	77.5 (35–100)
General health	75 (46–100)	86.8 (45–100)
Health change from the year before*	75 (0–100)*	50 (50–100)*

*One question where 50 = same health as last year, and >50 = better health than last year.

Each item is scored from 0 to 100 where 0 = poor health and 100= good health. Data are presented with median and total range values.

## Discussion

Nearly, two-fifths (39%) of the ski students entering a ski high school with an ACLR sustained an ACL reinjury within the two first years at the ski high school. The average age for an ACL reinjury was 16.5 years, and the average time from the first ACL injury was 18.7 months. However, 10 of the 12 ACL reinjuries occurred 10–23 months from the first ACL injury, which, on average, was 14.8 months from the first injury. The result is in line with previous research showing that an ACLR before the age of 20 combined with return to high level activity, predisposes an ACL reinjury (11, 16, 28, 29).

Skiing is an equilateral sport, which means that the ski turns have to be equally good to the left and to the right side, and consequently it is important to be equally good on both sides in functional performance tests. Previous research has shown a side-to-side difference to be a risk factor for an ACL injury in alpine skiing (8). This was also shown in this study where the ACL reinjury group had a larger side-to-side difference in the side hop test and in knee laxity measured with a KT-1000 arthrometer compared with those skiers who did not sustain an ACL reinjury. The side hop test may be considered a skiing-like hop test since the hop movement is performed from side to side. In this study, the difference between the groups was small, though significant, but maybe just the difference between mastering a ski turn equally good in both directions or not. Previous research has used limb symmetry index (LSI) to evaluate side-to-side differences between injured and uninjured legs in alpine skiing (30). However, in this study, where the students entered with an ACLR, this was not optional. In an injury profile study in ski high school students, the left leg was more injury prone than the right leg (4). One might speculate that, since most humans are right-sided, the left leg might be more vulnerable due to the equilateral demands of alpine skiing. Ten out of 12 ACL reinjuries in this study were in the left knee, and the reinjuries occurred more frequently in the ipsilateral knee compared with the contralateral knee, consequently more frequently to the left knee compared with the right knee. It could be of importance to understand whether a skier is right- or left-dominant in order to put special focus on the less dominant side with the aim to become equilateral.

Another highly important finding was based on the RAND 36-Item health survey all students entering the ski high school with an ACLR reported fairly low levels in the items energy/fatigue, pain, and general health compared with the other items and compared with normative data (31). Do the skiers return to skiing before fully recovered from their first injury? Nagelli and Hewett (17) suggested that return to unrestricted sports activity should be delayed until 2 years after an ACLR. This was based on biological and functional recovery. For example, the ligamentization process of the ACL autograft in humans may take up to 1 year for the patella-tendon autograft and 2 years for the hamstrings autograft (17). In addition, the neuromuscular system has to recover as well as bone and muscular strength, and sports-specific skills must be regained.

To recover and return to sports after an ACL injury is difficult. Considering the short time between the ACL injuries, one might suspect that the students were fighting with recovery from the first injury. In a study on gymnasts' experiences and perception of an ACL injury, the gymnasts described that early contact with a sports psychiatrist or likewise is important (30). They also described that one can become and feel stronger than ever, but if one does not prepare the body and mind for the specific gymnastics skills, one cannot succeed in the aim of returning to the previous level of gymnastics. Psychological readiness is a crucial part of returning to sports and may be the difference between whether the athlete returns to high-level sports or not (18, 19, 32, 33).

This study investigated students at ski high schools from year 1 to year 4. To attend a ski high school means leaving family behind and moving to a new place at a young age. In addition, these young students entered the school with a previous ACL injury and according to the health survey, with fairly low levels of energy, pain, and estimated general health. Maybe these alone are risk factors for an ACL reinjury, even if it was not shown in this study. In a study on young elite athletes, attending sports high school with a mean age of 17.1 years, it was found that sleeping 8 h or more and reaching recommended levels of nutrition intake reduces the odds to sustain an injury by more than 60% (34).

To prevent severe knee injuries in young competitive alpine skiers probably needs an algorithm including prevention based on risk factors and physical as well as psychological recovery in between training and competition. To date, only two prevention programs have been carried out on adolescent alpine skiers. In a study by Schoeb et al. (35), an off-snow injury prevention program for Swiss aged under 16 years (U16) competitive skiers performed once a week, reduced the 2-weekly prevalence of knee trauma, knee overuse, and lower back overuse complaints. Another study by Westin et al. (36) using a neuromuscular prevention program on- and off-snow, showed a reduction of first episode ACL injury in competitive alpine skiers between 16 and 20 years of age. Both these programs were carried out during the whole skiing season. In addition, psychological preparation must be included. This may be provided by the ski trainer, mental coach, medical team, family, or friends. For example, a young skier, attending a ski high school, does, in most cases, leave the secure environment at home for security provided by the school, friends, and the skiing community. Just to have someone to talk to, preferably someone with knowledge of alpine skiing, not involved in the regular ski training, could be one way of providing psychological preparation, security, and readiness to the skiers (32).

The fact that the students were followed prospectively from day 1 at the ski high school until they left the school 3–4 years later may be seen as a strength of this study. The students entered the school with a previous ACL injury, which made it possible to study ACL reinjuries. One limitation is that information about the first ACL injury is scarce, and the health status before the first ACL injury is unknown. Another limitation is the small sample size.

## Conclusion

Out of 31, 12 ski high school students (39%) entering the school with an ACLR sustained an ACL reinjury during the first 2 years at a ski high school and on average 18.7 months from the first ACL injury. Side-to-side differences were found in the side hop test and knee joint laxity, measured with the KT-1000 arthrometer, and may predispose an ACL reinjury in competitive adolescent alpine skiers. RAND 36-Item health survey did not predict an ACL reinjury.

## Data availability statement

The original contributions presented in the study are included in the article/supplementary materials, further inquiries can be directed to the corresponding author/s.

## Ethics statement

The studies involving human participants were reviewed and approved by Swedish Ethical Review Authority, Dnr 2006/833-31/1. Written informed consent from the participants' legal guardian/next of kin was not required to participate in this study in accordance with the national legislation and the institutional requirements.

## Author contributions

MW and MH have given substantial contribution to the conception, design of the study, and drafted the manuscript. Acquisition of data by MW. Analysis and interpretation by MW, LM, and MH. All authors critically revised, read, and approved the final version of the manuscript.

## Conflict of interest

The authors declare that the research was conducted in the absence of any commercial or financial relationships that could be construed as a potential conflict of interest.

## Publisher's note

All claims expressed in this article are solely those of the authors and do not necessarily represent those of their affiliated organizations, or those of the publisher, the editors and the reviewers. Any product that may be evaluated in this article, or claim that may be made by its manufacturer, is not guaranteed or endorsed by the publisher.
